# Treatment of Gingival Recession and Root Coverage Outcomes Using Fascia Lata Allograft: A Case Report with Two Years of Follow-Up

**DOI:** 10.1155/2024/9968705

**Published:** 2024-04-09

**Authors:** Fatima Liliana De Freitas Correia, Beatriz Raquel Yáñez-Ocampo, Cesar Augusto Esquivel Chirino, Daniela Carmona Ruiz, Delina Montes-Sánchez

**Affiliations:** ^1^Postgraduate Studies and Research Division, Periodontics and Implantology Department, School of Dentistry, National Autonomous University of Mexico, UNAM, Mexico City, Mexico; ^2^Department of Oral Biology, School of Dentistry, National Autonomous University of Mexico, UNAM, Mexico City, Mexico; ^3^Orthodontic Department, School of Dentistry, National Autonomous University of Mexico, UNAM, Mexico City, Mexico; ^4^Basic Biomedical Research, School of Stomatology, Campus Tehuacán, Benemerita Autonomous University of Puebla, Puebla City, Mexico

## Abstract

Mucogingival surgery is a procedure for the treatment of gingival recession, which is a shift of marginal gingival tissue to the cementoenamel junction (CEJ), exposing the surface of the root teeth. One treatment for gingival recession is the Langer and Langer bilaminar technique, which involves the use of the fascia lata (FL) membrane. This membrane is harvested from the aponeurosis of the external muscles. The purpose of this case report was to present the clinical results of a 2-year follow-up using the Langer and Langer bilaminar technique modified with FL in a patient with gingival recession. Recessions are a shift of marginal gingival tissue to the CEJ, which exposes the surface of the root teeth. At the 2-year follow-up, the patient presented with a gingival recession in tooth 41, which resulted in complete root coverage, reaching 83.3%; the amount of keratinized tissue increased to 3 mm in each tooth, changing the gingival biotype from thin to thick and scalloped. This case report supports the use of FL as a successful alternative treatment.

## 1. Introduction

Gingival recession is the movement of marginal gingival tissue to the enamel-cement junction (CEJ) and causes exposure to the surface of the root [1]. Gingival recession must be treated to eliminate hypersensitivity, abrasion, fracture, or root caries [2]. Root exposure can be corrected with surgical procedures that provide predictability based on the integrity of the interdental insertion, increasing the possibility of complete root coverage, while loss of interdental insertion reduces the possibility of complete root coverage [3]. To determine treatment and predict root coverage of gingival recession, the classification of the presence of the CEJ and the periodontal phenotype should be considered. A variety of surgical techniques are used to correct gingival recession and reduce root exposure. The type of gingival recession, presence of the CEJ, and presence of clinical periodontal symptoms determine the choice of technique [3, 4]. Different gum recession treatment techniques have varying success rates. Lateral displacement flaps have shown success rates ranging from 34 to 97% [5]. The average coverage of the coronally advanced flap was 97%, and the root coverage of the semilunar coronally positioned flap ranged from 90% to 95% [6, 7]. On the other hand, the tunnel technique has a success rate of 82% for covering localized gingival recessions and 87% for multiple recessions [8]. Vestibular incision subperiosteal tunnel access (VISTA) has an average root coverage of 88% for multiple recessions and a complete root coverage of 67% for multiple recessions [9]. The success rates ranged from 11% to 53% for gingival grafts [10]. The success rate of the subepithelial connective tissue graft technique ranged from 64.4% to 96.6% [11]. In contrast, deepithelialized connective tissue grafts showed an average root coverage of 80.3% [12]. A coverage of 98.4% is achieved with both connective tissue grafts and the tunnel technique [11]. With the use of biomaterials, root coverage with acellular dermal matrix (ADM) was 94% [13]. The xenogenic dermal matrix achieved 75.29% root coverage [14]. Finally, the FL membrane exhibited an average coverage rate of 94.2% [15]. The FL is used to cover roots and is characterized as viscous tissue, forming a functional three-dimensional collagen matrix that surrounds and infiltrates all body structures [16]. The fascial system serves to protect and support muscles and internal organs.

However, its main function is to reduce friction between muscles and the mechanical force generated by the muscle and skeletal system [17–19]. Comparisons between gingival recession treatments using FL versus autologous connective tissue grafts demonstrated a slight advantage for FL in covering all root surfaces compared to autologous grafts.

Comparison of the use of fascia lata membrane and autologous connective tissue graft for the coverage of gingival recessions at 6 months revealed a slight advantage in terms of the complete root coverage of the autologous graft (94.87 ± 0.14 mm) in comparison to the fascia lata membrane (94.24 ± 0.20 mm). These findings highlight the favorable clinical outcome of both transplants in the treatment of multiple GRs [15]. Therefore, the objective of this case report was to evaluate clinical outcomes after two years of follow-up using the Langer and Langer technique modified with FL.

## 2. Case Presentation

**Figure 1:**
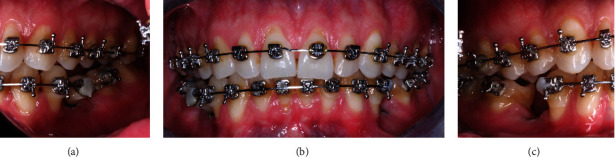
Intraoral photographs of the (a–c) central, left, and right areas.

**Figure 2:**
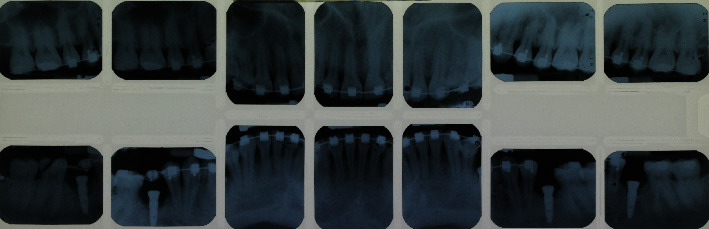
Periapical radiographs.

A 29-year-old Caucasian male presented to the National Autonomous University of Mexico Periodontics Department with no reported systemic disease or smoking. Tooth sensitivity was the reason for the consultation. During the clinical examination, parameters such as clinical attachment level, gum recession, dental biofilm index, and bleeding were documented. Inflammation in the marginal gingiva, the presence of supragingival calculus in the lower teeth, the loss of clinical attachment to the teeth 41 and 31 according to the Federal Dentaire International (FDI) numbering system, the absence of periodontal pockets, bleeding during examination, and the presence of orthodontic treatment were identified (Figures 1(a)–1(c) and 2). The patient consented by signing an informed consent form.

**Figure 3:**
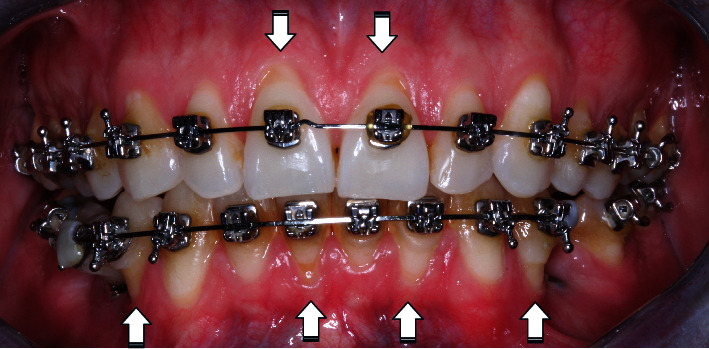
Gingival recessions (recessions are indicated by arrows).

**Figure 4:**
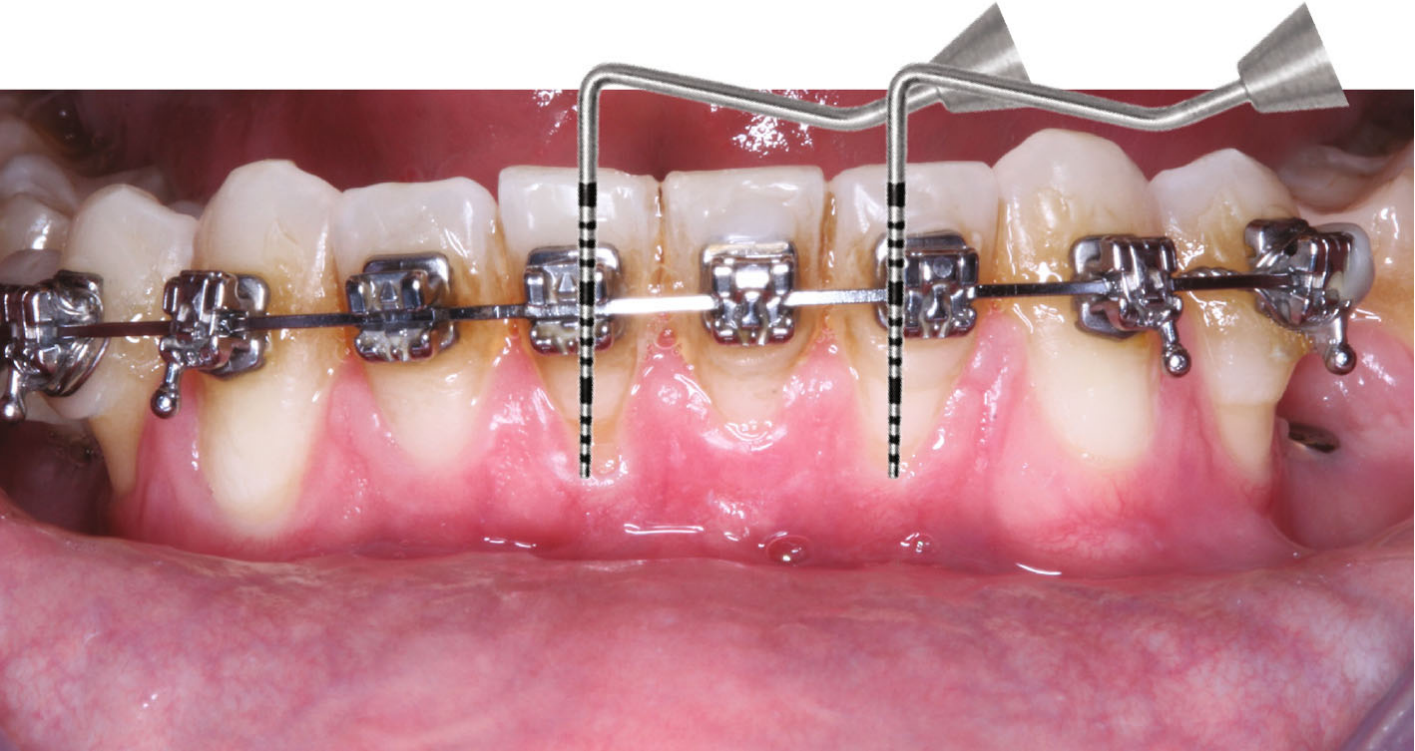
Gingival recession was measured with a periodontal probe.

The clinical diagnosis indicated plaque-induced gingivitis and gingival recession of RT1 de Cairo in teeth of types 11 (2 mm), 21 (1 mm), 41 (3 mm), and 32 (3 mm); gingival recession of RT 3 Cairo in teeth of types 34 (3 mm) and 44 (3 mm); and Seibert Class I ridge defects in teeth 35 and 45 [20]. According to the McGuire and Nunn classification, this disease has a “good” prognosis due to adequate periodontal support and control of etiologic factors [21] (Figures 3 and 4).

**Figure 5:**
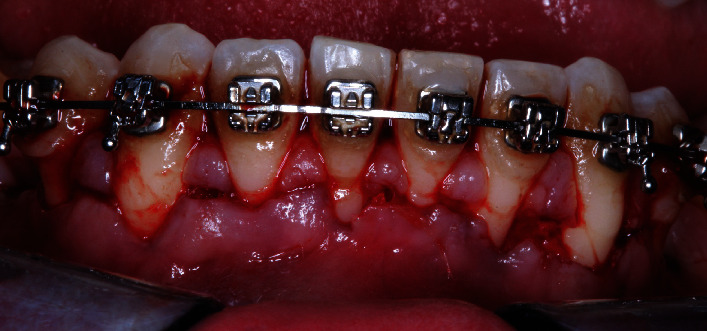
Horizontal and intrasulcular incisions.

Phase I periodontal procedures were performed, followed by Phase II procedures. This included root planning and scaling within recessions, treatment of recessions in teeth 41 and 32, resection and root planning of teeth 41 and 32, use of the Langer bilaminar technique modified [22] from teeth 33 to 43, and a right-angle incision of the coronal interproximal papillae (Figure 5).

**Figure 6:**
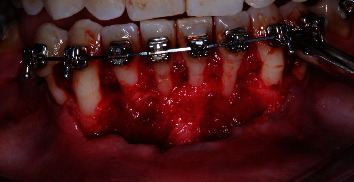
Partial thickness, full thickness, and partial thickness of the flap.

The FL was removed from the packaging and hydrated in sterile saline for 30 to 40 minutes. Intraoperative incisions were extended to the buccal aspect of each tooth with gingival recession to the adjacent tooth. After flap repositioning, the anatomical papillae that served as suture sites were deepithelialized with Lagrange surgical scissors. A partial-thickness flap was raised in the surgical papilla, while a full-thickness flap was raised to separate the inserted gingiva. A partial thickness flap was created at the mucogingival junction (MGJ) to release muscle attachments and allow passive positioning of the flap on all exposed root surfaces (Figure 6).

**Figure 7:**
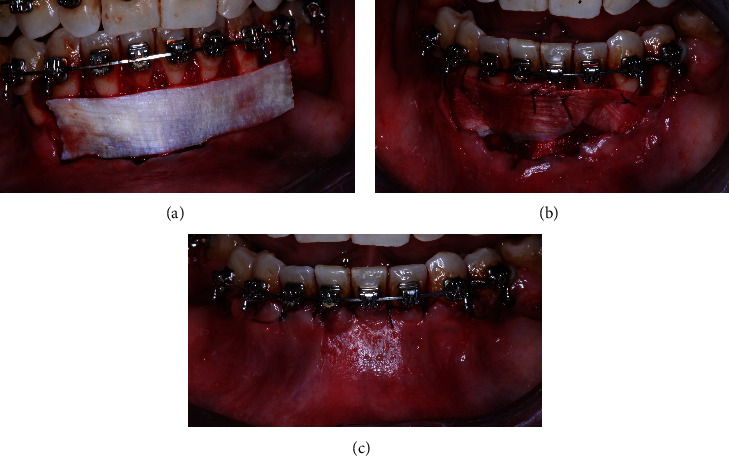
(a) Attachment of the recipient FL membrane to the receptor site, (b) attachment of the FL to the site, and (c) closure of the flap with suspension sutures.

The FL graft is secured on the recipient's bed with a 5-0 polyglycolic acid suture that passes through the interproximal soft tissue to ensure proper fixation of the graft and adequate blood supply. The flap completely covers the graft, is free, often extends at least 2 mm beyond the cementoenamel junction (CEJ), and is sutured with 5-0 polyglycolic acid (Figures 7(a)–7(c)). Patients are advised not to brush their teeth in the treated area and avoid abrupt movements that could injure the wound. For the first month, the patient was instructed to use a chlorhexidine rinse (0.12%) and take analgesics (400 mg ibuprofen every 8 hours for 3 days) twice daily.

## 3. Results

The results in this case report showed a coverage of 83.3% for teeth 41 and 32 after two years of postoperative evolution, using the formula presented by Zucchelli and De Sanctis [6]. The teeth (33, 31, 42, and 43) showed a change from thin to thick scalloped phenotypes.

(1) $$\begin{array}{c} 100\times \text{root coverage}*\text{Initial recession},\\ \text{Initial recession}*\left(\text{initial recession depth}-1‐\text{year follow}‐\text{up recession depth}\right). \end{array}$$

The initial measurements revealed gingival recession of 3 mm in tooth 41 and of 1 mm in tooth 31. The amount of keratinized tissue was 2 mm in each tooth, and the patient presented with a thin gingival thickness, detection of the CEJ in both teeth, superficial discrepancy in tooth 41, sensitivity, and aesthetic discrepancy. After two years of follow-up, the patient presented with gingival recession in tooth 41 (0.5 mm) and in tooth 31, the absence of gingival recession, the establishment of total root coverage in this tooth unit, the amount of keratinized tissue increased to 3 mm in each tooth unit, managing to change the gingival biotype, the absence of detection of CEJ in tooth 32, the absence of superficial discrepancy in tooth 41, and the absence of dental sensitivity and conformity aesthetics by the patient (Table 1).

## 4. Discussion

In this case report, we observed an average root coverage of 83.3% for teeth 41 and 32, which was achieved after a two-year postoperative period. According to Cairo et al., RT1 has a 100% prognosis for root coverage [3] in patients with adequately keratinized gingiva, average biotypes, and optimal oral hygiene, provided that they undergo regular monitoring to prevent healing changes.

The prediction of root coverage depends on the expertise of the operator and the surgical technique used. However, there is limited evidence regarding the efficacy of various treatments for improving root coverage. Several studies have shown that the coronally repositioned flap technique can yield average root coverage ranging from 63% to 86%, making it one of the most used mucogingival techniques for the treatment of the first and second recessions of the Cairo recessions, RT1 and RT [22].

It is essential to identify factors influencing clinical outcomes, including initial tooth thickness, which is an important determinant. Other factors include adjacent bone height, cartilage size, defect size, flap technique, and tooth location [22]. Pazos et al. reported the absorption percentages of FL and subepithelial connective tissue grafts. The first-month absorption rates were 28.8%, 53.48%, and 71.5%, and the second-month absorption rates were 43.47% and 61.69% for subepithelial tissue [23]. The initial thickness of the gingiva is critical for the successful treatment of exposed root surfaces and the achievement of complete root coverage with advanced coronal flaps. A gingival thickness greater than >1.2 ± 0.3 mm resulted in 100% root coverage, exceeding the <1.2 mm threshold [24] and compensating for the absorbed gingival tissue graft.

Autologous subepithelial connective tissue grafting is highly regarded for its favorable integration into the recipient tissue, ensuring reliable results, and is often considered the gold standard for restoring soft tissue defects in periodontal patients [15, 23, 25, 26]. However, it requires surgical intervention at two sites—the donor site and the recipient site—which increases postoperative patient morbidity. Additionally, the availability of autograft material is limited by anatomic and histologic constraints that limit the availability of donor tissue [27].

Anatomical constraints, such as the thickness, size, and depth of the patient's palate, represent inherent limitations beyond the control of the physician and directly affect the amount of tissue available [27] Autografting procedures are limited by the maximum tissue volume that can be obtained, making it impractical to address multiple gingival recessions in a single procedure [24].

“In several comparative studies, investigators have suggested that FL allografts and acellular dermal matrix serve as suitable alternatives to address the limitations associated with subepithelial connective tissue grafts, particularly in reducing postoperative morbidity and overcoming limitations related to available graft material [15, 23, 27, 28]. Bednarz et al. recognized the benefits of FL in patients unwilling to undergo surgery, thus reducing the need for extensive surgical procedures, especially in cases where adequate autologous graft material is limited [15]. An observed complication during the healing process in this patient was exposure to the fascia. Several studies have shown that when the fascia is exposed, it can be trimmed without complete removal, promoting favorable subsequent healing without additional complications [23]. In this case report, when exposure to the FL membrane was observed during follow-up, the protocol described by the authors was followed, resulting in the removal of the exposed membrane without complications.” Llano-Pérula et al. and Moeini et al. reported evidence on risk factors for gingival recession after orthodontic treatment in a systematic review. One was related to the thin periodontal phenotype, which is composed of a gingival phenotype (width of keratinized tissue 2.75-5.44 mm and height 0.63-1.24 mm) and a bone morphotype. The second risk factor was oral hygiene, which is directly related to the etiology of gingivitis and periodontitis, resulting in the importance of orthodontic-periodontic collaboration for interdisciplinary treatment to modify the gingival phenotype and control the etiology of periodontal disease with previous periodontal treatment before orthodontic treatment. In this case report, the patient had orthodontic treatment; therefore, orthodontic treatment is important for the treatment of gingival recession because properly aligning the teeth can redistribute the masticatory forces, reducing the pressure on the gum and preventing recession [28, 29].

Currently, there is limited evidence regarding the use of FL grafts in dental and periodontal treatments. The results from our use of FL were obtained from 1 patient; however, controlled trials using FL are needed.

## 5. Conclusion

The coverage of the roots treated with the FL membrane showed a favorable outcome two years after surgery. This suggests that the FL membrane serves as an alternative treatment for the correction of gingival recession in the anterior mandibular sector, particularly in thin scalloped phenotypes, resulting in a gain in root coverage comparable to that achieved with subepithelial connective tissue.

## Figures and Tables

**Table 1:** Classification of gingival phenotype, gingival recession, and tooth surface defects in areas of gingival recession.

Date	Tooth	RT	REC depth	GT	KTW	CEJ	Step
01/15/19	41	RT1	3 mm	Thin	2 mm	A	+
01/15/19	32	RT1	1 mm	Thin	2 mm	A	-
03/17/21	41	RT1	0.5 mm	Thick	3 mm	A	-
03/17/21	32	No recession	—	Thick	3 mm	B	-

RT = recession type; REC depth = depth of the gingival recession; GT = gingival thickness, KTW = keratinized tissue width; CEJ = cementoenamel junction (class A = detectable CEJ. Class B = undetectable CEJ); step = root surface concavity (class + = presence of a cervical step > 0.5 mm. Class - = absence of cervical step).

## Data Availability

The complete data used to support the diagnosis and findings of this case report are included within the article. The diagnostic records of the patient are available.
